# Mapping pathogenic processes contributing to neurodegeneration in *Drosophila* models of Alzheimer's disease

**DOI:** 10.1002/2211-5463.12773

**Published:** 2020-01-22

**Authors:** Liza Bergkvist, Zhen Du, Greta Elovsson, Hanna Appelqvist, Laura S. Itzhaki, Janet R. Kumita, Katarina Kågedal, Ann‐Christin Brorsson

**Affiliations:** ^1^ Division of Molecular Biotechnology Department of Physics, Chemistry and Biology Linköping University Sweden; ^2^ Department of Chemistry Centre for Misfolding Diseases University of Cambridge UK; ^3^ Department of Pharmacology University of Cambridge UK; ^4^ Department of Clinical and Experimental Medicine Faculty of Medicine and Health Sciences Linköping University Sweden; ^5^ Present address: Van Andel Research Institute 333 Bostwick Avenue NE Grand Rapids MI USA

**Keywords:** Alzheimer's disease, amyloid‐β, *Drosophila melanogaster*, endo‐lysosomal system, neurodegeneration

## Abstract

Alzheimer's disease (AD) is the most common form of dementia, affecting millions of people and currently lacking available disease‐modifying treatments. Appropriate disease models are necessary to investigate disease mechanisms and potential treatments. *Drosophila melanogaster* models of AD include the Aβ fly model and the AβPP‐BACE1 fly model. In the Aβ fly model, the Aβ peptide is fused to a secretion sequence and directly overexpressed. In the AβPP‐BACE1 model, human AβPP and human BACE1 are expressed in the fly, resulting in *in vivo* production of Aβ peptides and other AβPP cleavage products. Although these two models have been used for almost two decades, the underlying mechanisms resulting in neurodegeneration are not yet clearly understood. In this study, we have characterized toxic mechanisms in these two AD fly models. We detected neuronal cell death and increased protein carbonylation (indicative of oxidative stress) in both AD fly models. In the Aβ fly model, this correlates with high Aβ_1–42_ levels and down‐regulation of the levels of mRNA encoding lysosomal‐associated membrane protein 1, *lamp1* (a lysosomal marker), while in the AβPP‐BACE1 fly model, neuronal cell death correlates with low Aβ_1–42_ levels, up‐regulation of *lamp1* mRNA levels and increased levels of C‐terminal fragments. In addition, a significant amount of AβPP/Aβ antibody (4G8)‐positive species, located close to the endosomal marker rab5, was detected in the AβPP‐BACE1 model. Taken together, this study highlights the similarities and differences in the toxic mechanisms which result in neuronal death in two different AD fly models. Such information is important to consider when utilizing these models to study AD pathogenesis or screening for potential treatments.

## Graphical abstract

This study highlights the similarities and differences in the toxic mechanisms leading to neuronal death in two Drosophila models of Alzheimer's disease. Pathogenic processes investigated include expression levels of Aβ_1–42_, presence of AβPP C‐terminal fragments, extent of protein carbonylation (indicative of oxidative stress) and the effects on the endo‐lysosomal system determined by qPCR and immunohistochemistry.

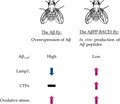

**Abbreviations**
AD: Alzheimer's disease
Aβ: amyloid beta
AβPP: amyloid beta precursor protein
BACE1: beta‐site AβPP‐cleaving enzyme
CTFs: C‐terminal fragments
MCI: mild cognitive impairment
TUNEL: terminal deoxynucleotidyl transferase dUTP nick end labelling

Alzheimer's disease (AD) is a neurodegenerative disorder that leads to progressive cognitive decline. It is the most prevalent form of dementia, affecting 11% of the population over the age of 65, and it is the sixth leading cause of death in the United States 1. A hallmark of the disease is the aggregation of the amyloid β (Aβ) peptide into fibrillar deposits known as amyloid plaques 2. However, research in the AD field points towards the soluble Aβ species, rather than the fibrillar deposits, as playing a key pathogenic role in the disease 3. The generation of Aβ peptides occurs through proteolytic processing of the transmembrane Aβ precursor protein (AβPP) by the β‐site AβPP‐cleaving enzyme (BACE1) followed by the intramembranous enzyme complex γ‐secretase 4, 5, 6. Depending on the site of cleavage, different‐sized Aβ peptides are generated, with Aβ_1–40_ and Aβ_1–42_ being the most frequent isoforms. Aβ_1–42_ has a higher propensity to form prefibrillar aggregates compared to Aβ_1–40_, and it has also been reported to be more toxic than Aβ_1–40_ 7. The Aβ peptides are not the only cleavage products from AβPP processing; when AβPP is first cleaved by BACE1, a C‐terminal fragment (CTF) consisting of 99 amino acids (C99) is produced. The level of C99 is higher in AD brains, and C99 from BACE1 cleavage of AβPP has been shown to overactivate rab5, leading to endosomal dysfunction 8.

To increase the understanding of the different pathways and mechanisms involved in AD, appropriate disease models are necessary. *Drosophila melanogaster*, the fruit fly, is one of the most well‐studied eukaryotes. The entire genome of the fruit fly was sequenced in 2000, and around 76% of human disease genes have homologues in the fly genome 9. For almost two decades, the fly has been used to study AD and Aβ proteotoxicity. The more commonly used Aβ fly model has the gene encoding the Aβ_1–42_ sequence cloned into the fly genome; the peptide is expressed fused to a signal sequence, resulting in secretion of the peptide to the extracellular space 10, 11, 12. In the other models, human AβPP is co‐expressed with human BACE1 allowing the production of C99 and different isoforms of the Aβ peptide (including post‐translationally modified Aβ variants) through the processing of human AβPP by human BACE1 and by endogenous fly γ‐secretase (the AβPP‐BACE1 fly model) 13, 14. AD fly models have been frequently used during the last two decades to investigate Aβ toxicity, cell‐specific vulnerability and aggregation 15, 16, 17, 18, 19, 20, 21, 22. However, potential differences in the toxic mechanisms between the two different AD fly models have not been thoroughly investigated. Recently, we published a study where the toxic effects in these two AD fly models were studied in parallel 14. We found that the proteotoxic effect, defined as the reduction in median survival time divided by total amount of Aβ_1–42,_ is considerably higher for the AβPP‐BACE1 flies compared to the Aβ_1–42_ flies, implying that the mechanisms of toxicity are different between these two AD fly models. In this study, we further investigate toxicity and disease mechanisms relevant in the context of AD for the Aβ_1–42_ and AβPP‐BACE1 flies by performing immunohistological and biochemical assays to probe: (a) the extent of neuronal death and protein carbonylation, (b) the gene expression level and distribution of markers of early endosomes and lysosomes and (c) the location of AβPP (and its cleavage products including Aβ_1–42_) and early endosomes and lysosomes in the fly CNS. Here, we present data which reveal that neuronal cell death is present in both AD fly models. The cell death was significantly higher in the Aβ_1–42_ flies compared to the AβPP‐BACE1 flies. However, the extent of cell death found in the AβPP‐BACE1 flies was remarkably high considering the low level of Aβ_1–42_ peptide detected in these flies (about 200 times lower than the Aβ_1–42_ flies). Therefore, to probe the pathological processes contributing to neuronal cell death in these two fly models, two cellular events that have been closely connected to AD, protein carbonylation and changes in the endo‐lysosomal system machinery were investigated 8, 23, 24, 25, 26.

## Results

### In both AD fly models, apoptosis leads to neuronal death

**Figure 1:**
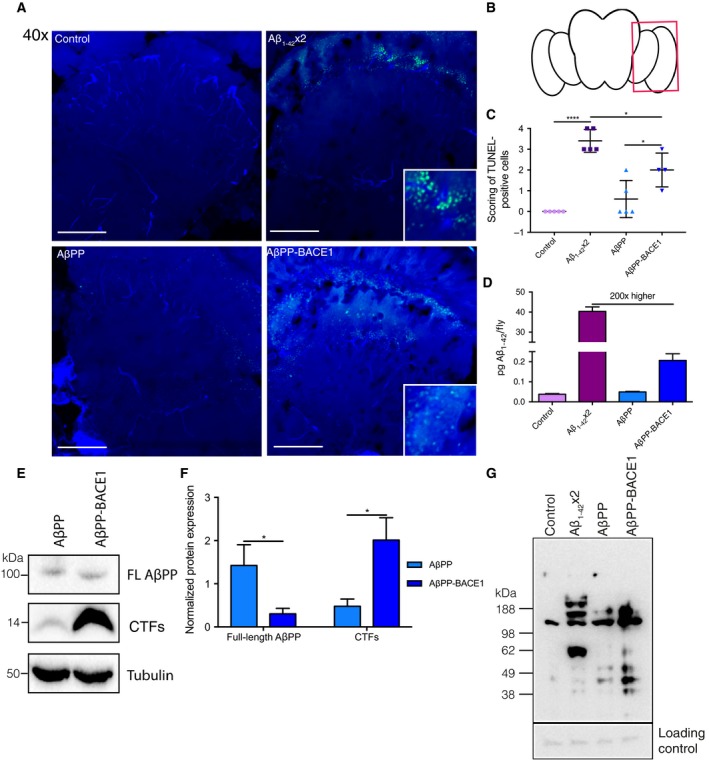
Both AD fly models demonstrate apoptotic cell death and protein carbonylation. (A) Apoptotic cells in control, Aβ_1–42_ × 2, AβPP and AβP‐BACE1 flies at day 21 identified by TUNEL (green) staining. Image inset highlights TUNEL‐positive cells. Micrographs were taken at 40× magnification, scale bar = 50 μm, *n* = 4–5 brains. DAPI was used to visualize cell nuclei (blue). (B) Schematic image of a fly brain where the red box indicates which areas were analysed for TUNEL‐positive cells; this corresponds to the medulla and the lamina. (C) Nonbiased scoring of the presence of TUNEL‐positive cells, *n* = 4–5, data represented as mean ± SD. * represents *P* ≤ 0.05 and **** represents *P* ≤ 0.0001 as determined by a one‐way ANOVA followed by Tukey's *post hoc* test. (D) Quantification of Aβ_1–42_ in the different fly genotypes at day 21, *n* = 3 (20 flies in each repeat). Data represented as mean ± SD. (E) Representative western blot showing the bands corresponding to full‐length AβPP and the CTFs for AβPP and AβPP‐BACE1 flies at day 21. Tubulin is used as a protein loading control, *n* = 4 (20 flies in each repeat). (F) Densitometry for full‐length AβPP and CTFs correlated to tubulin, data represented as mean ± SEM (*n* = 4). * represents *P* ≤ 0.05 as determined by the Mann–Whitney *U* test. (G) Representative immunoblot showing the total protein carbonylation in control, Aβ_1–42_ × 2, AβPP and AβPP‐BACE1 flies at day 21, *n* = 4 (20 flies in each repeat). Nonspecific band in nonderivatized negative control sample found in all sample preparations was used as a protein loading control.

Alzheimer's disease is the most common neurodegenerative disease; thus, neuronal cell death is a crucial feature of any potential AD animal model. By using the terminal deoxynucleotidyl transferase dUTP nick end labelling (TUNEL) assay, the presence of apoptotic cells in brain sections from *Drosophila* was investigated for control w^1118^ (only expressing Gal4), AβPP (human AβPP_695_), Aβ_1–42_ × 2 (fly line with two copies of Aβ_1–42_) and AβPP‐BACE1 (human AβPP_695_ and human BACE1) flies (Fig. 1A). Flies were analysed at day 21, a time point corresponding to the median survival time previously observed for AβPP‐BACE1 flies 14. The majority of all TUNEL‐positive cells were observed in the medulla and the lamina (Fig. 1B). By scoring the presence of TUNEL‐positive cells in a blind fashion, a significant increase in the number of TUNEL‐positive cells was observed for both the Aβ_1–42_ × 2 (*P* ≤ 0.0001) and the AβPP‐BACE1 (*P* ≤ 0.05) flies relative to their control flies (w^1118^ and AβPP flies, respectively), demonstrating the presence of apoptotic cells in both model systems (Fig. 1C). The increase in TUNEL‐positive cells was significantly higher (*P* ≤ 0.05) for the Aβ_1–42_ × 2 flies compared to the AβPP‐BACE1 flies, revealing a higher level of neuronal apoptosis in the Aβ_1–42_ × 2 flies at the selected time point.

### The Aβ_1–42_ load is significantly higher in the Aβ_1–42_ × 2 flies compared to the AβPP‐BACE1 flies

As the Aβ_1–42_ peptide is closely linked to AD and neurodegeneration 24, 27, 28, the total level of Aβ_1–42_ present in the different fly genotypes was determined (Fig. 1D). The highest level of Aβ_1–42_ was detected in the Aβ_1–42_ × 2 flies (40 ± 2.6 pg per fly), which was approximately 200 times higher than the level detected in the AβPP‐BACE1 flies (0.20 ± 0.04 pg per fly). Thus, a significantly higher level of Aβ_1–42_ is present in the Aβ_1–42_ × 2 flies compared to the AβPP‐BACE1 flies and this correlates with the higher level of neuronal apoptosis observed in the Aβ_1–42_ × 2 flies compared to the AβPP‐BACE1 flies.

### Increased level of the C‐terminal fragments in the AβPP‐BACE1 flies compared to the AβPP flies

After the first cleavage of full‐length AβPP by BACE1 or by fly intrinsic α‐secretase, two different CTFs are produced (C99 and C83, respectively), and C99 from BACE1 cleavage of AβPP may be involved in neurotoxic events 8. To specifically investigate the presence of full‐length AβPP and CTFs in the AβPP flies and the AβPP‐BACE1 flies, a western blot was performed using a C‐terminal AβPP antibody from Sigma‐Aldrich (St. Louis, MO, USA) (Fig. 1E – entire blot in Fig. S1). The result revealed a significant decrease in the level of full‐length AβPP and a significant increase in the level of CTFs (C99) in the AβPP‐BACE1 flies compared to the AβPP flies (Fig. 1F).

### Increased protein carbonylation in both AD fly models

Mitochondrial dysfunction and subsequent increased oxidative stress have been connected with neurodegeneration and AD 23. Protein carbonylation, an indicator of oxidative stress 29, was investigated in the fly models. Protein carbonylation was detected in all four genotypes (Fig. 1G); however, an increase in protein carbonylation was detected for both the Aβ_1–42_ × 2 flies and the AβPP‐BACE1 flies compared to their respective controls (w^1118^ and AβPP flies). Interestingly, the proteins that were carbonylated differed between the Aβ_1–42_ × 2 and AβPP‐BACE1 flies. In the Aβ_1–42_ × 2 flies, two carbonylated protein bands were detected, one band above 188 kDa and one band around 62 kDa. These two bands were essentially absent in the AβPP‐BACE1 flies, and the carbonylation detected in the AβPP‐BACE1 flies occurred for proteins with lower molecular weights compared to the Aβ_1–42_ × 2 flies (< 62 kDa).

### Distribution of early endosomes and lysosomes in the two AD fly models

**Figure 2:**
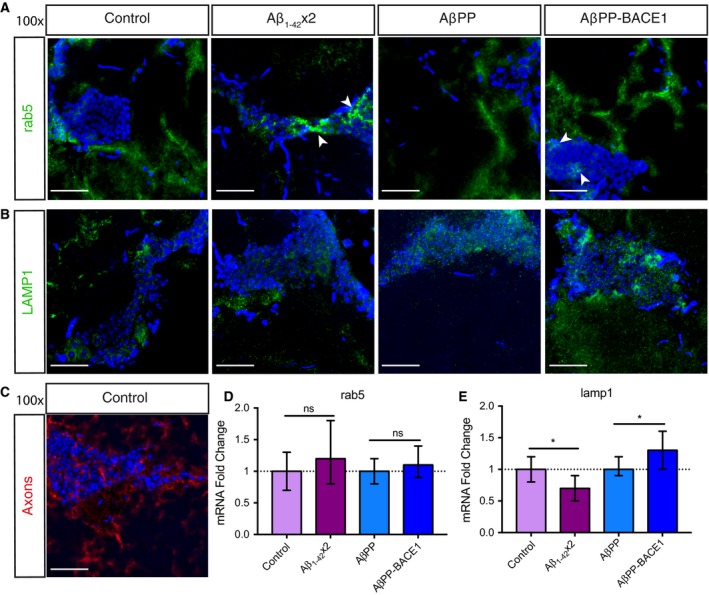
Lysosomal alterations in AD fly models. (A) *Drosophila* brain sections, day 21, of control, Aβ_1–42_ × 2, AβPP and AβPP‐BACE1 flies were stained with a *Drosophila* anti‐rab5 antibody (marker for early endosomes, green) or (B) with a *Drosophila* anti‐LAMP1 antibody (marker for lysosomes, green). DAPI (blue) was used to visualize cell nuclei. White arrowheads indicate perinuclear rab5 staining in Aβ_1–42_ × 2 and AβPP‐BACE1 flies in panel (A). Micrographs were taken at 100× magnification, scale bar = 20 μm and *n* = 6 in (A) and (B). (C) *Drosophila* brain sections of control flies stained with a *Drosophila* anti‐axon antibody, *n* = 3. mRNA levels of *rab5* (D) and *lamp1* (E) were analysed, *n* = 3 (20 flies in each repeat). * represent *P* ≤ 0.05 as determined by Wilcoxon signed‐rank test. The final data presented as $2^{\Delta \Delta C_{\min}}$ to $2^{\Delta \Delta C_{\max}}$ with SE.

Endosomal and lysosomal dysfunctions can be observed in the early stages of AD, and with time, it progresses to a widespread failure of intraneuronal waste clearance and eventually neuronal death 26, 30, 31, 32. To investigate the distribution of early endosomes and lysosomes in the AD flies, *Drosophila* brain sections for control w^1118^, AβPP, Aβ_1–42_ × 2 and AβPP‐BACE1 flies were stained with a *Drosophila* anti‐rab5 antibody, investigating the presence of early endosomes (Fig. 2A), or with a *Drosophila* anti‐LAMP1 antibody, investigating the presence of lysosomes (Fig. 2B). The area of the brain analysed is the same as for the TUNEL analysis, highlighted in Fig. 1B.

The immunohistochemistry analysis showed that early endosomes were located perinuclear as well as separated from the cell bodies in all fly genotypes (Fig. 2A). Staining control w^1118^ flies with a *Drosophila* anti‐axon antibody reveals a network of axons separated from the cell bodies (Fig. 2C). This staining pattern of axons is very similar to the staining pattern of early endosomes separated from the cell nuclei. Thus, the early endosomes detected separated from the cell bodies are likely located in this network of axons, indicating that early endosomes are present both around the cell nuclei, in the cell body and in the axons of the fly neurons. No significant differences in the *rab5* mRNA levels were observed between the four genotypes (Fig. 2D).

The immunohistochemistry analysis of the distribution of lysosomes showed both perinuclear staining and staining separated from the cell bodies in all fly genotypes (Fig. 2B). Looking at the mRNA level of the lysosomal marker, LAMP1, a small but significant (*P* ≤ 0.05) up‐regulation of *lamp1* was detected for the AβPP‐BACE1 flies compared to AβPP flies while a small but significant (*P* ≤ 0.05) down‐regulation was detected for *lamp1* mRNA in the Aβ_1–42_ × 2 flies compared to control w^1118^ flies (Fig. 2E).

Taken together, the distribution of endosomes and lysosomes was found both perinuclear and separated from the cell bodies. No differences in the mRNA levels of the *rab5* endosomal marker were detected, but an up‐regulation of *lamp1* was observed in the AβPP‐BACE1 flies compared to AβPP flies, whereas there was a down‐regulation in *lamp1* mRNA in the Aβ_1–42_ × 2 flies compared to control w^1118^ flies.

### The AβPP/Aβ antibody 4G8 signal occurs in close vicinity to the staining pattern of early endosomes in the AβPP‐BACE1 flies

**Figure 3:**
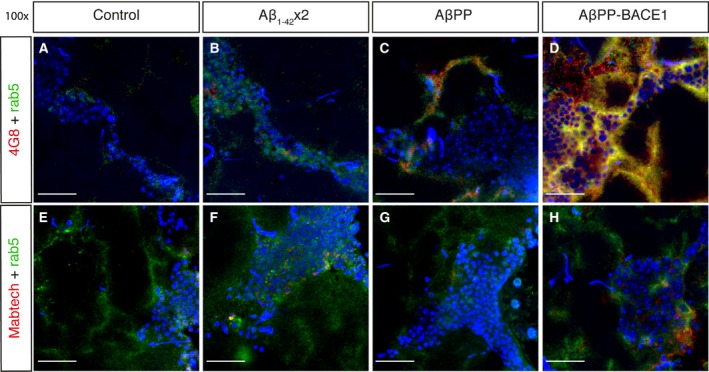
AβPP/Aβ antibody 4G8 signal occurs in the vicinity of early endosomes in the AβPP‐BACE1 flies. *Drosophila* brain sections (day 21) of control, Aβ_1–42_ × 2, AβPP and AβPP‐BACE1 flies costained with a *Drosophila* anti‐rab5 antibody (green; early endosomes), and the AβPP/Aβ antibody 4G8 (red) (A–D) or the N‐terminal Aβ antibody from Mabtech (red) (E–H). DAPI was used to visualize cell nuclei (blue). Micrographs were taken at 100× magnification, scale bar = 20 μm and *n* = 6.

To compare the location of AβPP and/or Aβ with early endosomes, *Drosophila* brain sections were costained with the *Drosophila* anti‐rab5 antibody and the AβPP/Aβ antibody 4G8 (which is known to react to both the Aβ peptide and full‐length AβPP 33) or the N‐terminal Aβ antibody from Mabtech (Nacka Strand, Sweden) (Fig. 3). The area of the brain analysed is the same as for the TUNEL analysis, highlighted in Fig. 1B. Control w^1118^ flies showed no 4G8 or Mabtech staining (Fig. 3A,E). In the Aβ_1–42_ × 2 flies, the 4G8 and Mabtech signals were located around the cell nuclei (Fig. 3B,F). In the AβPP flies, a 4G8 signal was detected in the axons, separated from the cell bodies and in close vicinity to the staining pattern of early endosomes (Fig. 3C). No Mabtech signal was detected in the AβPP flies (Fig. 3G). In the AβPP‐BACE1 flies, an intense 4G8 signal was present both around the cell nuclei and in the axons, in close vicinity to the staining pattern of early endosomes (Fig. 3D). A Mabtech signal was observed in the AβPP‐BACE1 flies around the cell nuclei (Fig. 3H).

**Figure 4:**
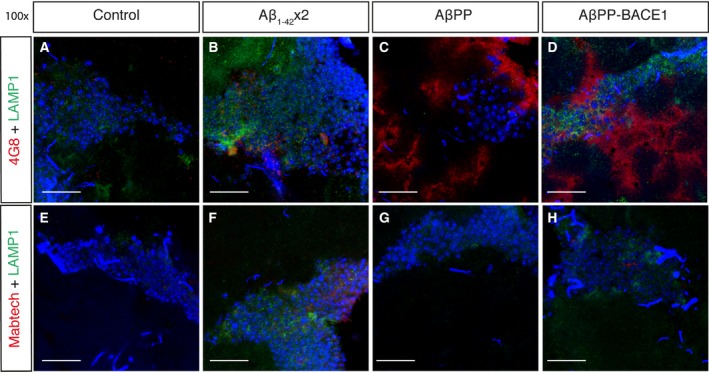
Signals for the AβPP/Aβ antibody 4G8 nor Aβ antibody Mabtech do not coincide with lysosomes in the AD fly models. *Drosophila* brain sections (day 21), of control, Aβ_1–42_ × 2, AβPP and AβPP‐BACE1 flies were costained with a *Drosophila* anti‐LAMP1 antibody (green; lysosomal marker), to investigate the presence of lysosomes, and the AβPP/Aβ antibody 4G8 (red) (A–D) or with the N‐terminal Aβ antibody from Mabtech (red) (E–H). DAPI was used to visualize cell nuclei (blue). Micrographs were taken at 100× magnification, scale bar = 20 μm and *n* = 6.

Next, *Drosophila* brain sections were costained with the *Drosophila* anti‐LAMP1 antibody and 4G8 or the Mabtech antibody to compare the locations of AβPP and/or Aβ and lysosomes in the fly brain (Fig. 4). Control w^1118^ flies showed no 4G8 or Mabtech staining (Fig. 4A,E). As observed in the previous staining (Fig. 3B,F), the 4G8 and Mabtech signals were located around the cell nuclei in the Aβ_1–42_ × 2 flies (Fig. 4B,F). In the AβPP flies, a 4G8 signal was located in the axons, separated from the cell bodies, but no lysosome staining occurred at this location (Fig. 4C). No Mabtech signal was detected in the AβPP flies (Fig. 4G). In the AβPP‐BACE1 flies, an intense 4G8 signal was present around the cell nuclei and in the axons but the signal did not coincide with the lysosome staining (Fig. 4D). A Mabtech signal was observed in the AβPP‐BACE1 flies around the cell nuclei (Fig. 4H).

Taken together, a signal from the 4G8 antibody was detected around the cell nuclei for the Aβ_1–42_ × 2 flies, in the axons for the AβPP flies and in both places for the AβPP‐BACE1 flies. The staining pattern of 4G8 and endosomes coincided in the AβPP flies and the AβPP‐BACE1 flies, while the 4G8 signal in the Aβ_1–42_ × 2 did not coincide with the endosome signal. The staining pattern of lysosomes did not coincide with the 4G8 signal in any of the flies. Signals from the Mabtech antibody were observed around the cell nuclei for the Aβ_1–42_ × 2 and for the AβPP‐BACE1 flies but did not coincide with the lysosome or endosome signals.

## Discussion

Understanding the underlying mechanisms of AD toxicity is a key requirement to developing mechanism‐based therapeutic strategies, and the use of *Drosophila* to investigate the pathogenesis of AD has allowed scientists to achieve important goals in this research field 34. AD research using *Drosophila* frequently implies one of two approaches; either the Aβ peptides are fused to a secretion sequence and directly produced from transgenes (the Aβ fly model) or the Aβ peptides are produced by the processing of human AβPP (the AβPP‐BACE1 fly model) 10, 14, 35, 36, 37, 38. In this paper, we have looked, in detail, at the pathways leading to toxicity within the two AD fly models and have highlighted differences in the underlying mechanisms of the AD‐related toxicity observed in these systems.

In our previous study, longevity and locomotor analyses showed significant toxic effects for both the Aβ42 flies and AβPP‐BACE1 flies 14. The time frame selected for this study was 21 days, corresponding to the median survival time for the AβPP‐BACE1 flies. Around this age, the flies in both AD models start to display dysfunctional locomotor behaviour. Studies have shown that dysfunctional locomotor behaviour in *Drosophila* is associated with neurodegeneration 39. The results from the TUNEL assay revealed the presence of apoptotic cells in both AD models albeit to a higher extent in the Aβ_1–42_ × 2 flies compared to the AβPP‐BACE1 flies. This difference in apoptotic cell death was found to correlate with the dramatically higher level of Aβ_1–42_ present in the Aβ_1–42_ × 2 flies, where 200 times more Aβ_1–42_ accumulated as compared to the AβPP‐BACE1 flies at day 21. This difference in the Aβ_1–42_ level is in concordance with previous data demonstrating a ratio of 1:40 of the Aβ_1–42_ level between the AβPP‐BACE1 and the Aβ_1–42_ × 2 flies at day 7 14. Hence, Aβ_1–42_ accumulates to an even higher degree in the Aβ_1–42_ × 2 flies compared to the AβPP‐BACE1 flies with subsequent ageing.

The Aβ_1–42_ peptide is more hydrophobic than the shorter isoforms and is therefore more prone to aggregating and forming toxic species 7, 40, 41. It can form large amyloid aggregates which can sequester other proteins, leading to toxicity due to loss of function 42. Aβ_1–42_ oligomers of different sizes have been found to impair memory in AD rodent models and the peptide itself has been shown to interact with other proteins, such as cell surface receptors, leading to downstream signalling which may contribute to neurodegeneration 43, 44, 45, 46. Thus, it is likely that the neuronal death observed in the Aβ_1–42_ × 2 flies is due to high accumulation of toxic Aβ_1–42_ species. Indeed, this is supported by several other studies where high levels of Aβ_1–42_ have been shown to cause neurodegeneration in *Drosophila* models of AD 12, 47, 48.

An early event in AD pathology is an increase in oxidative stress, which can be observed in patients with mild cognitive impairment (MCI) before any significant increase in amyloid plaques or neurofibrillary tangles can be detected 23. Oxidative stress is an indicator of mitochondrial dysfunction, causing a rise in reactive oxygen species which results in an increase in protein carbonylation 29. Interestingly, both the Aβ_1–42_ × 2 flies and the AβPP‐BACE1 flies showed an increase in protein carbonylation compared to control w^1118^ and AβPP flies. This implies that oxidative stress is a possible contributor to neurodegeneration in both AD fly models. The Aβ peptide has been shown to impair degradation of mitochondrial proteins and to change mitochondrial membrane potential, which may trigger the release of cytochrome c and thus induce apoptosis 25, 49, 50. Therefore, a noticeable contribution to the neuronal death in the AβPP‐BACE1 flies could be due to intracellular Aβ that disrupts mitochondrial function, leading to increased oxidative stress and eventually apoptosis. This can explain how a relatively low level of Aβ_1–42_ may induce neurodegeneration.

Another early event in AD pathology includes abnormalities in the endo‐lysosomal pathway 30 where increased levels of rab5 and rab7 proteins, markers for early and late endosomes, respectively, have been found to be up‐regulated in individuals with MCI as well as in AD patients 32. Aβ has been shown to accumulate in lysosomes, a pathogenic event indicating a loss of lysosomal integrity and the ability to degrade its material 51, 52, 53, 54. Endo‐lysosomal pathways are essential in maintaining cellular homeostasis. Dysfunction of this intriguing system has been suggested to represent a converging mechanism for many diseases involving neurodegeneration, including AD 55. Investigation of the endo‐lysosomal system in the two AD fly models revealed that *Lamp1* mRNA was increased in the AβPP‐BACE 1 flies and decreased in the Aβ_1–42_ × 2 flies. The increased *Lamp1* mRNA level in the AβPP‐BACE flies is in line with previous studies where increased *lamp1* mRNA expression in AβPPSL transgenic mice expressing AβPP with Swedish and London mutations has been found 56. These data suggest abnormalities in the endo‐lysosomal system for both fly models that might contribute to the toxicity in these flies. For the AβPP‐BACE1 flies, abnormality in the endo‐lysosomal system may explain toxicity despite the low level of Aβ_1–42_ in these flies. Indeed, small amounts of intracellular accumulation of Aβ in endocytic vesicles can trigger Aβ oligomerization 57, disrupting the vesicles' ability to mature and leading to a decrease in protein degradation and eventually inducing toxicity. For the Aβ_1–42_ flies, the toxicity may be caused by the down‐regulation of lysosomes resulting in the lysosome machinery being overwhelmed by Aβ species and consequently leading to neuronal death.

BACE1 is able to cleave AβPP at the plasma membrane, but more frequently, BACE1 cleavage occurs in the early endosomes resulting in the production of C99 58. Interestingly, Aβ is not the only cleavage product from AβPP processing known to cause endosomal dysfunction; C99 produced from BACE1 cleavage of AβPP has been shown to pathologically activate rab5, leading to an accumulation of swollen endosomes 8. In both the AβPP and AβPP‐BACE1 flies, the signal for the AβPP/Aβ antibody (4G8) was detected in close vicinity with *Drosophila* endosomes. Interestingly, the coincidence of these signals was distributed in different areas within the two flies. In the AβPP flies, the area where the 4G8 and endosome signals coincide is located distinctly from the cell nuclei in the axons, while in the AβPP‐BACE1 flies, the 4G8 and endosome signals were strongly clustered around the cell nuclei as well as in the axons. The Mabtech signal (specific for the Aβ peptide) in the AβPP‐BACE1 flies did not coincide with the endosome staining, suggesting that the 4G8 signal in the AβPP‐BACE1 flies corresponds to either full‐length AβPP or C99. The increase in the C99 level detected for the AβPP‐BACE1 flies compared to the AβPP flies suggests that the 4G8 staining around the cell nuclei in the AβPP‐BACE1 flies corresponds to accumulation of C99 while the 4G8 staining visible in the axons of the AβPP‐BACE1 flies and the AβPP flies corresponds to full‐length AβPP. Thus, the high level of C99 detected for the AβPP‐BACE1 flies that coincided with endosomes, together with the increased amount of apoptotic cells identified in these flies, compared to the AβPP flies, suggests that a possible contributor to the apoptosis in the AβPP‐BACE1 flies is the accumulation of C99 in endosomal vesicles. This may lead to a disruption in the endosomal pathway that will decrease the ability of the neurons to degrade or recycle proteins, thereby leading to apoptosis 26. In the Aβ_1–42_ × 2 flies, the 4G8 and Mabtech signals did not coincide with either endosome or lysosome markers, despite being in close proximity to the cell nuclei. Hence, if these species, detected by 4G8 and Mabtech antibodies, are located intracellularly, they are generally not associated with endosomes or lysosomes. Another possibility is that the 4G8 and Mabtech signals in the Aβ_1–42_ × 2 flies are detecting aggregated extracellular Aβ species. Indeed, both the 4G8 and Mabtech antibodies have been documented to detect not only monomeric Aβ but also oligomers and larger aggregated species 33.

Taken together, in this study we have identified possible toxic mechanisms in two distinct AD fly models; high levels of Aβ_1–42_ correlate with a high number of apoptotic cells in the Aβ_1–42_ × 2 flies, which also displays increased protein carbonylation indicating oxidative stress. In addition, the lysosomal machinery was found to be slightly down‐regulated in the Aβ_1–42_ × 2 flies which can contribute to the pathological events detected in this model. In the AβPP‐BACE1 flies, a considerable amount of apoptotic cells was detected, and these flies also display increase in protein carbonylation, representative of oxidative stress. However, it is unlikely that the small amount of Aβ_1–42_ detected is solely responsible for the cell death in these flies. Possible contributors to the toxicity in the AβPP‐BACE1 flies are an increased level of intracellular C99 and abnormalities in the endo‐lysosomal system.

Notably, this study highlights the versatility of these fly models and how they can be used to increase our understanding of the mechanisms underlying AD. Furthermore, taken together, these AD fly models present a possibility to investigate potential treatment strategies that target Aβ production and Aβ aggregation but also other cellular events closely linked to the disease, for example oxidative stress and dysfunction in the endo‐lysosomal pathway.

## Materials and methods

### *Drosophila* stocks

The Gal4/UAS system was used to achieve a tissue‐specific protein expression in UAS transgenic *D. melanogaster* 59. *Elav*‐Gal4 flies were used as the driver line. This allows expression in the CNS and the PNS, in developing neuronal cells and in early glial cells of the flies. Control w^1118^ flies (only expressing Gal4) were used as a control for the Aβ_1–42_ × 2 flies, and a fly line expressing Gal4 and human AβPP was used as a control for the AβPP‐BACE1 flies. The AβPP‐BACE1 fly model has previously been described 14. Aβ_1–42_ flies were kindly provided by D. Crowther (AstraZeneca, Floceleris, Oxbridge Solutions Ltd.). These Aβ flies produce an aberrant Aβ42 peptide with additional N‐terminal glutamine residue 19. A fly line containing double copies of signal peptide Aβ_1–42_ (Aβ_1–42 _× 2 flies) was generated as previously described 48. The fly lines were not backcrossed prior to the experiment. Fly crosses were set up at 18 °C at 60% humidity with 12:12‐h light:dark cycles. For all biochemical assays, flies were aged for 21 days at 29 °C and then snap‐frozen or embedded in Tissue‐Tek OCT Compound (25608‐930; VWR, Stockholm, Sweden).

### Samples preparation and protein quantification of Aβ_1–42_

For the analysis of total Aβ_1–42_, a multispot 96‐well V‐PLEX human Aβ_1–42_ kit plate (K151LBE‐1; Meso Scale Discovery, Rockville, MD, USA) was used. Samples were prepared, and quantification was carried out as previously described in Ref 14. In short, approximately 20 fly heads or bodies were homogenized in 25 μL extraction buffer [50 mm HEPES, 5 m guanidinium chloride, 5 mm EDTA, 1 × protease inhibitor (cOmplete EDTA‐free Protease Inhibitor Cocktail Tablets; Roche Diagnostics, Basel, Switzerland)], for extraction of both insoluble and soluble Aβ_1–42_ species. After correcting total protein concentration in each sample due to differences in the homogenization step using the Bio‐Rad DC Protein Assay Kit II (500‐0112; Bio‐Rad, Hercules, CA, USA), protein samples were added to the wells of a multispot 96‐well V‐PLEX human Aβ_1–42_ kit plate. The assay was then carried out according to manufacturer's instructions.

### TUNEL assay

OCT blocks with embedded fly heads were sectioned using a Microm HM550 Cryostat (Microm International GmbH, Dreieich, Germany) into 20‐μm‐thin sections and stored at −20 °C until use. The TUNEL assay was performed using FragEL™ DNA Fragmentation Detection Kit, Fluorescent – TdT Enzyme (QIA39; Merck Millipore, Burlington, MA, USA). The assay was carried out as per the manufacturer's instructions; however, the incubation time with proteinase K was set to 2 min and the sections were allowed to incubate with the TdT enzyme for 60 min at 37 °C. The slides were analysed using a Zeiss LSM 780 confocal microscope (Zeiss, Oberkochen, Germany). Micrographs were processed in Adobe Photoshop (Adobe Systems, San Jose, CA, USA); background levels were reduced, and the signal levels were enhanced. All images were treated identically. For each genotype, four to five brain sections corresponding to the medulla and lamina were scored in a nonbiased fashion. The scoring system ranged from 0 (no TUNEL‐positive cells), 1 (a few TUNEL‐positive), 2 (more TUNEL‐positive cells, but still a lot of TUNEL‐negative cells), 3 (approximately 50% TUNEL‐positive cells) to 4 (more TUNEL‐positive cells than TUNEL‐negative cells). The data were plotted and analysed using graphpad prism 7 (San Diego, CA, USA). To identify any significant difference between the groups, a one‐way ANOVA followed by Tukey's *post hoc* test was performed.

### qPCR analysis

w^1118^, Aβ_1–42_ × 2, AβPP and AβPP‐BACE1 flies were collected and stored at −80 °C. Total RNA was extracted using the RNeasy Micro Plus Kit (Qiagen, Caldwell, ID, USA). The A260/A280 was determined to be above 2.0 on a NanoDrop ND2000 UV‐vis Spectrophotometer (Labtech International Ltd., Uckfield, UK), and the RNA integrity was confirmed on a 1% agarose gel showing a single band ~ 2.0 kbp in size, representative of the 18S rRNA and the 28S rRNA (which, in *Drosophila*, is cleaved into two fragments that migrate at the same position as the 18S rRNA) 60. cDNA was synthesized using the RNA samples and the ImProm‐II™ Reverse Transcription System (Promega UK Ltd., Southampton, UK). qPCR primer sequences for the *Drosophila* genes, *rab5* and *lamp1*, and the reference genes, *gapdh2* and *αTub84B*, were previously published 61. Standard curves for all four genes were generated using cDNA concentrations of 0.04, 0.2, 1, 5 and 25 ng and performing standard qPCRs under the experimental conditions: a 20 μL reaction included 0.2 μm primers (Sigma‐Aldrich), Fast SYBR^®^ Green Master Mix (Thermo Fisher Scientific, Waltham, MA, USA), cDNA (ranging 0.04–25 ng per well) and dH_2_O. Efficiency of all reactions was found to be between 90 and 110%, and therefore, the use of the comparative *C*_T_ method for data analysis was applied 62. Reactions were performed in a StepOnePlus Real‐Time PCR System (Applied Biosystems Ltd., Foster City, CA, USA). Each well included: 0.2 µm primer, 2.5 ng cDNA and 1× Fast SYBR^®^ Green Master Mix; each sample was analysed in duplicate. Reactions were performed with an initial denaturation (95 °C, 10 min), followed by 42 cycles of denaturation (95 °C, 15 s), annealing and extension (60 °C, 1 min). Melting curves were monitored between 60 °C and 95 °C. Products were checked by electrophoresis on a 2% agarose gel to verify the presence of one single band (amplicon) with a correct product size. Data were collected from three independent batches (*n* = 3) of flies (20 flies in each repeat). The qPCR results from multiple runs were analysed using the comparative *C*_T_ method 62. The change in expression of the two target genes (*rab5* and *lamp1*) in AβPP‐BACE1 (Aβ_1–42_ × 2) was determined relative to the appropriate control sample, that is AβPP (w^1118^), and presented as mRNA fold change. Wilcoxon signed‐rank test was used to test statistical significance.

### Immunohistochemistry

OCT blocks with embedded fly heads were sectioned as described above. The sections were fixed with 4% (w/v) PFA for 10 min at RT and then washed 3 × 3 min with PBS‐T. Additional permeabilization of the sections was carried out using 0.5% Tween‐20 for 10 min at RT. The washing step was repeated, and the sections were blocked for 60 min at RT using 10% BSA in PBS‐T. After blocking, the sections were incubated with the primary antibodies, 4G8 (SIG‐39220; BioLegend, San Diego, CA, USA); anti‐human Amyloid‐β mAb Abeta (3740‐5‐250; Mabtech); anti‐rab5 antibody (ab31261; Abcam, Cambridge, UK); anti‐LAMP1 antibody (ab30687); anti‐axons antibody (ab12455), all diluted 1 : 500 in 1% BSA in PBS‐T, incubated overnight at 4 °C. After repeating the washing step, the sections were incubated with secondary antibodies goat anti‐mouse Alexa 594 (R37121; Thermo Fisher Scientific) and goat anti‐rabbit Alexa 488 (R37116; Thermo Fisher Scientific), diluted 1 : 500 in 1% BSA for 60 min at RT. After a final washing step, the sections were rinsed with dH_2_O and left to dry before mounting them with VECTASHIELD DAPI (H‐1200; Vector Laboratories, Burlingame, CA, USA). The slides were analysed using a Zeiss LSM 780 confocal microscope. Micrographs were processed in Adobe Photoshop; background levels were reduced, and the signal levels were enhanced. All images were treated identically.

### Protein carbonylation assay

The heads of snap‐frozen flies (20 flies/genotype) were homogenized in 25 μL RIPA lysis and extraction buffer (89900; Thermo Fisher Scientific) with 1× Protease Inhibitor (cOmplete EDTA‐free Protease Inhibitor Cocktail Tablets; Roche Diagnostics) and 50 mm dithiothreitol. After centrifuging the samples for 10 min at 18 928 ***g***, the supernatant was collected and the total protein level extracted was determined using a Bio‐Rad DC Protein Assay Kit II (500–0112; Bio‐Rad). Samples were prepared to have a final protein concentration of approx. 30 mg·mL^−1^. The sample preparation was then divided into two Eppendorf tubes, where derivatization of the carbonyl groups was carried out using the OxyBlot Protein Oxidation Detection Kit (S7150; Merck, Kenilworth, NJ, USA) according to the manufacturer's instructions on one half of the sample. The other half was used as a negative control, where derivatization‐control solution (S7150; Merck) was added instead of DNPH solution (S7150; Merck). Gel electrophoresis was performed using Bolt 4–12% Bis‐Tris Plus Gels (NW04120BOX; Life Technologies, Carlsbad, CA, USA). Transfer was performed using an original iBlot^®^ Gel Transfer Device from Life Technologies onto PVDF mini membranes (IB401002; Life Technologies). The membrane was blocked using 10% BSA for 1 h at RT. The primary antibody (rabbit anti‐DNP antibody, 90451; Merck) was prepared diluted 1 : 150 in 1% BSA and added to the membrane for 1 h, RT. This was followed by a washing step, 3 × 3 min with PBS‐T before adding the secondary antibody (goat anti‐rabbit, HRP‐conjugated, 90452; Merck) for 1 h, RT, diluted 1 : 300 in 1% BSA. The washing step was repeated before incubating the membrane with Clarity Western ECL Substrate (1705060S; Bio‐Rad) for 5 min before imaging on a ImageQuant LAS 4000 (GE Healthcare Life Sciences, Marlborough, MA, USA). Bands from the nonderivatized negative control sample preparation that appears in all samples were used as a loading control.

### Western blot analysis

Protein extract from fly heads was obtained as described above. Samples of approximately 5 µg) were loaded onto a Bolt 12% Bis‐Tris Plus Gel and after protein separation by electrophoresis transferred onto a nitrocellulose membrane. The membrane was boiled for 5 min in PBS and thereafter blocked in 5% milk in TBS‐Tween. Immunodetection was performed with monoclonal primary antibodies: anti‐C‐terminal AβPP (A8717, 1 : 8000; Sigma‐Aldrich) and antitubulin (loading control; ab7291; 1 : 2000; Abcam) followed by HRP‐conjugated corresponding secondary antibodies (Dako, Santa Clara, CA, USA). Densitometric analysis was performed on four separate blots using imagej 1.50i (Wayne Rasband, National Institutes of Health, Bethesda, MD, USA). Bands corresponding to full‐length APP and the C‐terminal cleavage fragment (CTF) were normalized to tubulin expression. Statistical analysis was performed using the Mann–Whitney *U* test. Differences were considered significant when *P* ≤ 0.05.

## Conflict of interest

The authors declare no conflict of interest.

## Author contributions

A‐CB and LB conceived and designed the project; LB, ZD, HA and GE acquired the data; LB, A‐CB, ZD, JRK, LSI, HA and KK analysed and interpreted the data; A‐CB, LB, KK, ZD, HA, JRK and LSI wrote the paper.

## Supporting information


**Fig. S1.** Entire blot containing the specific bands for full length AβPP and CTFs shown in Fig. 1E.

## References

[1] Thies W and Bleiler L (2013) 2013 Alzheimer's disease facts and figures. Alzheimers Dement 9, 208–245. 23507120 10.1016/j.jalz.2013.02.003

[2] Hardy JA and Higgins GA (1992) Alzheimer's disease: the amyloid cascade hypothesis. Science 256, 184–185. 1566067 10.1126/science.1566067

[3] Esparza TJ , Wildburger NC , Jiang H , Gangolli M , Cairns NJ , Bateman RJ and Brody DL (2016) Soluble amyloid‐beta aggregates from human Alzheimer's disease brains. Sci Rep 6, 1–16. 27917876 10.1038/srep38187 PMC5137165

[4] De Strooper B and Annaert W (2000) Proteolytic processing and cell biological functions of the amyloid precursor protein. J Cell Sci 1, 1857–1870. 10.1242/jcs.113.11.1857 10806097

[5] Kandalepas PC , Sadleir KR , Eimer WA , Zhao J , Nicholson DA and Vassar R (2013) The Alzheimer's beta‐secretase BACE1 localizes to normal presynaptic terminals and to dystrophic presynaptic terminals surrounding amyloid plaques. Acta Neuropathol 126, 329–352. 23820808 10.1007/s00401-013-1152-3 PMC3753469

[6] Zhang X and Song W (2013) The role of APP and BACE1 trafficking in APP processing and amyloid‐β generation. Alzheimers Res Ther 5, 46. 24103387 10.1186/alzrt211 PMC3978418

[7] Dahlgren KN , Manelli AM , Blaine Stine W , Baker LK , Krafft GA and Ladu MJ (2002) Oligomeric and fibrillar species of amyloid‐beta peptides differentially affect neuronal viability. J Biol Chem 277, 32046–32053. 12058030 10.1074/jbc.M201750200

[8] Kim S , Sato Y , Mohan PS , Peterhoff C , Pensalfini A , Rigoglioso A , Jiang Y and Nixon RA (2016) Evidence that the rab5 effector APPL1 mediates APP‐βCTF‐induced dysfunction of endosomes in Down syndrome and Alzheimer's disease. Mol Psychiatry 21, 707–716. 26194181 10.1038/mp.2015.97 PMC4721948

[9] Reiter LT , Potocki L , Chien S , Gribskov M and Bier E (2001) A systematic analysis of human disease‐associated gene sequences in *Drosophila melanogaster* . Genome Res 11, 1114–1125. 11381037 10.1101/gr.169101 PMC311089

[10] Crowther DC , Page R , Chandraratna D and Lomas DA (2006) A *Drosophila* model of Alzheimer's disease. Methods Enzymol 412, 234–255. 17046662 10.1016/S0076-6879(06)12015-7

[11] Finelli A , Kelkar A , Song HJ , Yang H and Konsolaki M (2004) A model for studying Alzheimer's Abeta42‐induced toxicity in *Drosophila melanogaster* . Mol Cell Neurosci 26, 365–375. 15234342 10.1016/j.mcn.2004.03.001

[12] Iijima K , Liu H‐P , Chiang A‐S , Hearn SA , Konsolaki M and Zhong Y (2004) Dissecting the pathological effects of human Aβ40 and Aβ42 in Drosophila: a potential model for Alzheimer's disease. Proc Natl Acad Sci USA 101, 6623–6628. 15069204 10.1073/pnas.0400895101 PMC404095

[13] Greeve I , Kretzschmar D , Tschäpe J‐A , Beyn A , Brellinger C , Schweizer M , Nitsch RM and Reifegerste R (2004) Age‐dependent neurodegeneration and Alzheimer‐amyloid plaque formation in transgenic *Drosophila* . J Neurosci 24, 3899–3906. 15102905 10.1523/JNEUROSCI.0283-04.2004 PMC6729409

[14] Bergkvist L , Sandin L , Kågedal K and Brorsson A‐C (2016) AβPP processing results in greater toxicity per amount of Aβ 1–42 than individually expressed and secreted Aβ 1–42 in *Drosophila melanogaster* . Biol Open 5, 1030–1039. 27387531 10.1242/bio.017194 PMC5004604

[15] Jonson M , Nyström S , Sandberg A , Carlback M , Michno W , Hanrieder J , Starkenberg A , Nilsson KPR , Thor S and Hammarström P (2018) Aggregated Aβ1‐42 is selectively toxic for neurons, whereas glial cells produce mature fibrils with low toxicity in *Drosophila* . Cell Chem Biol 25, 595–610.e5. 29657084 10.1016/j.chembiol.2018.03.006

[16] Lee S , Bang SM , Hong YK , Lee JH , Jeong H , Park SH , Liu QF , Lee IS and Cho KS (2016) The calcineurin inhibitor Sarah (Nebula) exacerbates Aβ42 phenotypes in a *Drosophila* model of Alzheimer's disease. Dis Model Mech 9, 295–306. 26659252 10.1242/dmm.018069 PMC4826976

[17] Sowade RF and Jahn TR (2017) Seed‐induced acceleration of amyloid ‐β mediated neurotoxicity in vivo. Nat Commun 8, 1–12. 28894090 10.1038/s41467-017-00579-4 PMC5594032

[18] Ott S , Dziadulewicz N and Crowther DC (2015) Iron is a specific cofactor for distinct oxidation‐ and aggregation‐dependent A toxicity mechanisms in a *Drosophila* model. Dis Model Mech 8, 657–667. 26035384 10.1242/dmm.019042 PMC4486857

[19] Allan K , Perez KA , Barnham KJ , Camakaris J and Burke RA (2014) Commonly used *Drosophila* model of Alzheimer’s disease generates an aberrant species of amyloid‐β with an additional N‐terminal glutamine residue. FEBS Lett 588, 3739–3743. 25171862 10.1016/j.febslet.2014.08.022

[20] Ray A , Speese SD and Logan MA (2017) Glial draper rescues Aβ toxicity in a *Drosophila* model of Alzheimer's disease. J Neurosci 37, 11881–11893. 29109235 10.1523/JNEUROSCI.0862-17.2017 PMC5719972

[21] Rodin DI , Schwarzman AL , Sarantseva SV and Division RB (2015) Expression of human amyloid precursor protein in *Drosophila melanogaster* nerve cells causes a decrease in presynaptic gene mRNA levels. Genet Mol Res 14, 9225–9232. 26345855 10.4238/2015.August.10.2

[22] Arnés M , Casas‐Tintó S , Malmendal A and Ferrús A (2017) Amyloid β42 peptide is toxic to non‐neural cells in *Drosophila* yielding a characteristic metabolite profile and the effect can be suppressed by PI3K. Biol Open 6, 1664–1671. 29141953 10.1242/bio.029991 PMC5703620

[23] Wang X , Wang W , Li L , Perry G , Lee H‐G and Zhu X (2013) Oxidative stress and mitochondrial dysfunction in Alzheimer's disease. Biochim Biophys Acta 1842, 1240–1247. 24189435 10.1016/j.bbadis.2013.10.015 PMC4007397

[24] Hoozemans JJM , Chafekar SM , Baas F , Eikelenboom P and Scheper W (2006) Always around, never the same: pathways of amyloid beta induced neurodegeneration throughout the pathogenic cascade of Alzheimer's disease. Curr Med Chem 13, 2599–2605. 17017913 10.2174/092986706778201585

[25] Mossmann D , Vögtle F‐N , Taskin AA , Teixeira PF , Ring J , Burkhart JM , Burger N , Pinho CM , Tadic J , Loreth D * et al* (2014) Amyloid‐β peptide induces mitochondrial dysfunction by inhibition of preprotein maturation. Cell Metab 20, 662–669. 25176146 10.1016/j.cmet.2014.07.024

[26] Nixon RA (2017) Amyloid precursor protein & endosomal‐lysosomal dysfunction in Alzheimer's disease: inseparable partners in a multifactorial disease. FASEB J 31, 2729–2743. 28663518 10.1096/fj.201700359 PMC6137496

[27] Walsh DM and Selkoe DJ (2007) Aβ oligomers – a decade of discovery. J Neurochem 101, 1172–1184. 17286590 10.1111/j.1471-4159.2006.04426.x

[28] Sadigh‐Eteghad S , Sabermarouf B , Majdi A , Talebi M , Farhoudi M and Mahmoudi J (2015) Amyloid‐beta: a crucial factor in Alzheimer's disease. Med Princ Pract 24, 1–10. 10.1159/000369101 PMC5588216 25471398

[29] Dalle‐Donne I , Rossi R , Giustarini D , Milzani A and Colombo R (2003) Protein carbonyl groups as biomarkers of oxidative stress. Clin Chim Acta 329, 23–38. 12589963 10.1016/s0009-8981(03)00003-2

[30] Cataldo AM , Peterhoff CM , Troncoso JC , Gomez‐Isla T , Hyman BT and Nixon RA (2000) Endocytic pathway abnormalities precede amyloid beta deposition in sporadic Alzheimer's disease and Down syndrome: differential effects of APOE genotype and presenilin mutations. Am J Pathol 157, 277–286. 10880397 10.1016/s0002-9440(10)64538-5 PMC1850219

[31] Adamec E , Mohan PS , Cataldo AM , Vonsattel JP and Nixon RA (2000) Up‐regulation of the lysosomal system in experimental models of neuronal injury: implications for Alzheimer's disease. Neuroscience 100, 663–675. 11098128 10.1016/s0306-4522(00)00281-5

[32] Ginsberg S , Alldred M and Counts S (2010) Microarray analysis of hippocampal CA1 neurons implicates early endosomal dysfunction during Alzheimer's disease progression. Biol Psychiatry 68, 885–893. 20655510 10.1016/j.biopsych.2010.05.030 PMC2965820

[33] Hunter S and Brayne C (2017) Do anti‐amyloid beta protein antibody cross reactivities confound Alzheimer disease research? J Negat Results Biomed 16, 1–8. 28126004 10.1186/s12952-017-0066-3 PMC5270220

[34] Moloney A , Sattelle DB , Lomas DA and Crowther DC (2010) Alzheimer's disease: insights from *Drosophila melanogaster* models. Trends Biochem Sci 35, 228–235. 20036556 10.1016/j.tibs.2009.11.004 PMC2856915

[35] Caesar I , Jonson M , Nilsson KPR , Thor S and Hammarström P (2012) Curcumin promotes A‐beta fibrillation and reduces neurotoxicity in transgenic *Drosophila* . PLoS ONE 7, e31424. 22348084 10.1371/journal.pone.0031424 PMC3278449

[36] Sandin L , Bergkvist L , Nath S , Kielkopf C , Janefjord C , Helmfors L , Zetterberg H , Blennow K , Li H , Nilsberth C *et al* (2016) Beneficial effects of increased lysozyme levels in Alzheimer's disease modelled in *Drosophila melanogaster* . FEBS J 283, 3508–3522. 27562772 10.1111/febs.13830 PMC5132093

[37] Chakraborty R , Vepuri V , Mhatre SD , Paddock BE , Miller S , Michelson SJ , Delvadia R , Desai A , Vinokur M , Melicharek DJ * et al* (2011) Characterization of a *Drosophila* Alzheimer's disease model: pharmacological rescue of cognitive defects. PLoS ONE 6, e20799. 21673973 10.1371/journal.pone.0020799 PMC3108982

[38] Mhatre SD , Satyasi V , Killen M , Paddock BE , Moir RD , Saunders AJ and Marenda DR (2014) Synaptic abnormalities in a *Drosophila* model of Alzheimer's disease. Dis Model Mech 7, 373–85. 24487408 10.1242/dmm.012104 PMC3944497

[39] Ali YO , Escala W , Ruan K and Zhai RG (2011) Assaying locomotor, learning, and memory deficits in *Drosophila* models of neurodegeneration. J Vis Exp 49, 2504. 10.3791/2504 PMC3197301 21445036

[40] El‐Agnaf OMA , Mahil DS , Patel BP and Austen BM (2000) Oligomerization and toxicity of β‐amyloid‐42 implicated in Alzheimer's disease. Biochem Biophys Res Commun 273, 1003–1007. 10891362 10.1006/bbrc.2000.3051

[41] Sengupta U , Nilson AN and Kayed R (2016) The role of amyloid‐β oligomers in toxicity, propagation, and immunotherapy. EBioMedicine 6, 42–49. 27211547 10.1016/j.ebiom.2016.03.035 PMC4856795

[42] Olzscha H , Schermann SM , Woerner AC , Pinkert S , Hecht MH , Tartaglia GG , Vendruscolo M , Hayer‐Hartl M , Hartl FU and Vabulas RM (2011) Amyloid‐like aggregates sequester numerous metastable proteins with essential cellular functions. Cell 144, 67–78. 21215370 10.1016/j.cell.2010.11.050

[43] Lesné S , Koh MT , Kotilinek L , Kayed R , Glabe CG , Yang A , Gallagher M and Ashe KH (2006) A specific amyloid‐beta protein assembly in the brain impairs memory. Nature 440, 352–357. 16541076 10.1038/nature04533

[44] Walsh DM , Klyubin I , Fadeeva JV , Cullen WK , Anwyl R , Wolfe MS , Rowan MJ and Selkoe DJ (2002) Naturally secreted oligomers of amyloid beta protein potently inhibit hippocampal long‐term potentiation in vivo. Nature 416, 535–539. 11932745 10.1038/416535a

[45] Snyder EM , Nong Y , Almeida CG , Paul S , Moran T , Choi EY , Nairn AC , Salter MW , Lombroso PJ , Gouras GK *et al* (2005) Regulation of NMDA receptor trafficking by amyloid‐beta. Nat Neurosci 8, 1051–1058. 16025111 10.1038/nn1503

[46] Magdesian MH , Carvalho MMVF , Mendes FA , Saraiva LM , Juliano MA , Juliano L , Garcia‐Abreu J and Ferreira ST (2008) Amyloid‐β binds to the extracellular cysteine‐rich domain of frizzled and inhibits Wnt/β‐catenin signaling. J Biol Chem 283, 9359–9368. 18234671 10.1074/jbc.M707108200 PMC2431018

[47] Speretta E , Jahn TR , Tartaglia GG , Favrin G , Barros TP , Imarisio S , Lomas DA , Luheshi LM , Crowther DC and Dobson CM (2012) Expression in *Drosophila* of tandem amyloid β peptides provides insights into links between aggregation and neurotoxicity. J Biol Chem 287, 20748–20754. 22461632 10.1074/jbc.M112.350124 PMC3370257

[48] Crowther DC , Kinghorn KJ , Miranda E , Page R , Curry JA , Duthie FA , Gubb DC and Lomas DA (2005) Intraneuronal Abeta, non‐amyloid aggregates and neurodegeneration in a *Drosophila* model of Alzheimer's disease. Neuroscience 132, 123–135. 15780472 10.1016/j.neuroscience.2004.12.025

[49] Abramov AY (2004) Amyloid peptides induce mitochondrial dysfunction and oxidative stress in astrocytes and death of neurons through activation of NADPH oxidase. J Neurosci 24, 565–575. 14724257 10.1523/JNEUROSCI.4042-03.2004 PMC6729998

[50] Kim HS , Lee JH , Lee JP , Kim EM , Chang KA , Park CH , Jeong SJ , Wittendorp MC , Seo JH , Choi SH *et al* (2002) Amyloid beta peptide induces cytochrome c release from isolated mitochondria. NeuroReport 13, 1989–1993. 12395106 10.1097/00001756-200210280-00032

[51] De Kimpe L , Van Haastert ES , Kaminari A , Zwart R , Rutjes H , Hoozemans JJM and Scheper W (2013) Intracellular accumulation of aggregated pyroglutamate amyloid beta: convergence of aging and Aβ pathology at the lysosome. Age 35, 673–687. 22477259 10.1007/s11357-012-9403-0 PMC3636379

[52] Zheng L , Cedazo‐Minguez A , Hallbeck M , Jerhammar F , Marcusson J and Terman A (2012) Intracellular distribution of amyloid beta peptide and its relationship to the lysosomal system. Transl Neurodegener 1, 19. 23210724 10.1186/2047-9158-1-19 PMC3514139

[53] Domert J , Rao SB , Agholme L , Brorsson AC , Marcusson J , Hallbeck M and Nath S (2014) Spreading of amyloid‐β peptides via neuritic cell‐to‐cell transfer is dependent on insufficient cellular clearance. Neurobiol Dis 65, 82–92. 24412310 10.1016/j.nbd.2013.12.019

[54] Ling D , Magallanes M and Salvaterra PM (2014) Accumulation of amyloid‐like Aβ_1–42_ in AEL (autophagy–endosomal–lysosomal) vesicles: potential implications for plaque biogenesis. ASN Neuro 6, 95–109. 10.1042/AN20130044 PMC4379859 24521233

[55] Wang C , Telpoukhovskaia MA , Bahr BA , Chen X and Gan L (2018) Endo‐lysosomal dysfunction: a converging mechanism in neurodegenerative diseases. Curr Opin Neurobiol 48, 52–58. 29028540 10.1016/j.conb.2017.09.005

[56] Hashimoto T , Ogino K , Shin RW , Kitamoto T , Kikuchi T and Shimizu N (2010) Age‐dependent increase in lysosome‐associated membrane protein 1 and early‐onset behavioral deficits in APPSL transgenic mouse model of Alzheimer's disease. Neurosci Lett 469, 273–277. 20025930 10.1016/j.neulet.2009.12.015

[57] Oddo S , Caccamo A , Tran L , Lambert MP , Glabe CG , Klein WL and LaFerla FM (2006) Temporal profile of amyloid‐beta (Abeta) oligomerization in an in vivo model of Alzheimer disease: a link between Abeta and tau pathology. J Biol Chem 281, 1599–1604. 16282321 10.1074/jbc.M507892200

[58] Vassar R (1992) Beta‐secretase cleavage of Alzheimer's amyloid precursor protein by the transmembrane aspartic protease BACE. Science 286, 735–741. 10.1126/science.286.5440.735 10531052

[59] Brand AH and Perrimon N (1993) Targeted gene expression as a means of altering cell fates and generating dominant phenotypes. Development 118, 401–415. 8223268 10.1242/dev.118.2.401

[60] Pellegrini M , Manning J and Davidson N (1977) Sequence arrangement of the rDNA of *Drosophila melanogaster* . Cell 10, 213–214. 402223 10.1016/0092-8674(77)90215-x

[61] Ling D and Salvaterra PM (2011) Robust RT‐qPCR data normalization: validation and selection of internal reference genes during post‐experimental data analysis. PLoS ONE 6, e17762. 21423626 10.1371/journal.pone.0017762 PMC3058000

[62] Schmittgen TD and Livak KJ (2008) Analyzing real‐time PCR data by the comparative C(T) method. Nat Protoc 3, 1101–1108. 18546601 10.1038/nprot.2008.73

