# Supplementary figures and images for: Mapping pathogenic processes contributing to neurodegeneration in Drosophila models of Alzheimer's disease
Source: FEBS Open Bio. 2020 Jan 22;10(3):338–50. doi: 10.1002/2211-5463.12773 (PMC7050262; doi:10.1002/2211-5463.12773)

$A\beta_{PP}$   
 $A\beta_{PP}$ -BACE1

100 kDa

– full length  $A\beta_{PP}$

14 kDa

– CTFs

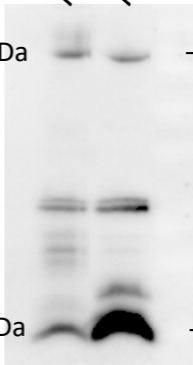

Supplement: Supplementary file 1 — Fig. S1. Entire blot containing the specific bands for full length AβPP and CTFs shown in Fig. 1E. [file FEB4-10-338-s001.pdf]
